# Online survey data of public subjective well-being on high occupancy vehicle lane in China

**DOI:** 10.1016/j.dib.2017.10.039

**Published:** 2017-10-26

**Authors:** Shunxi Li, Bowen Su, Pang-Chieh Sui, Guofang Zhang

**Affiliations:** a School of Automotive Engineering, Wuhan University of Technology, Hubei 430070, China; b Hubei Key Laboratory of Advanced Technology for Automotive Components and Hubei Collaborative Innovation Center for Automotive Components Technology, Hubei 430070, China

## Abstract

The data presented in this article are related to the research article entitled “Out-of-home activities, daily travel, and SWB” (Ettema et al., 2010) [1]. The paper provides an online survey questionnaire and data about the public subjective well-being of high occupancy vehicle lanes in China. The survey data are made publicly available to extended analysis.

**Specifications table**

**Table:** 

Subject area	*Psychology, Transport*
More specific subject area	*Subjective well-being, High occupancy vehicle lane*
Type of data	*Table*
How data was acquired	*Survey*
Data format	*Raw*
Experimental factors	*Gender, age, travel mode, city size*
Experimental features	*An online survey was carried out on the online questionnaire website in China between May and October, 2016*
Data source location	*China*
Data accessibility	*The data are available with this article*

**Value of the data**

The questionnaire and survey data of investigation from online survey may be used to design and improve the high occupancy vehicle lanes of the city, and to help to research the carpooling policy of developing countries based on the subjective well-being on high occupancy vehicle lane of different genders or ages.

## 1 Data

Table 1 shows the demographics of the 695 survey takers. Table 2 is the online survey data of subjective well-being on high occupancy vehicle lane between different ages. Table 3 is the online survey data of subjective well-being on high occupancy vehicle lane between male and female. Table 4 shows the survey factors and corresponding question numbers in part II of questionnaire. The Appendix A gives the questionnaire used in the study.

**Table 1:** Demographics of the survey takers.

Feature	Option	N	Rate
Gender	Male	325	46.8%
	Female	370	53.2%
Age	Under 18	25	3.6%
	18–30	445	64.0%
	30–45	210	30.2%
	Over 45	15	2.2%
Travel mode	On foot	105	15.1%
	Bus	235	33.8%
	Subway	90	13.0%
	Private car	75	32.4%
	Others	40	5.8%
Travel purpose	Work	375	54.0%
	Daily shopping	130	18.7%
	Entertainment	75	10.8%
	Pick up children	95	13.7%
	Others	20	2.9%
City scale	First-tier city	155	22.3%
	Second-tier city	275	39.6%
	Others	265	38.1%
Heard or used HOV lane before	Yes	450	64.8%
	No	245	35.3%

**Table 2:** Online survey data of subjective well-being on high occupancy vehicle lane between different ages.

	Components	Factors	Age options	Mean	Standard deviation
Subjective	Affective factors	Safety	1	1.90	0.285
well-being			2	2.15	0.755
on high			3	2.54	0.646
occupancy			4	1.75	1.090
vehicle lane		Fairness	1	2.10	0.418
			2	2.30	0.649
			3	2.36	0.670
			4	1.67	0.946
		Comfort	1	2.05	0.326
			2	2.22	0.694
			3	2.48	0.632
			4	1.75	0.500
		Enjoyment	1	2.00	0.637
			2	2.01	0.694
			3	2.39	0.816
			4	2.25	0.661
	Instrumental factors	Efficiency	1	1.47	0.381
			2	2.09	0.753
			3	2.57	0.853
			4	1.78	0.387
		Reliability	1	1.80	0.605
			2	2.04	0.701
			3	2.50	0.780
			4	1.67	0.665
		Size^a^	1	2.00	0.817
			2	2.15	0.832
			3	2.56	0.832
			4	1.89	0.840

a Size refers to the length, number and coverage of high occupancy vehicle lanes in a city.

**Table 3:** Online survey data of subjective well-being on high occupancy vehicle lane between male and female.

	Components	Factors	Gender	Mean	Standard deviation
Subjective well-being on high occupancy vehicle lane	Affective factors	Safety	Male	2.08	0.694
			Female	2.41	0.750
		Fairness	Male	2.22	0.612
			Female	2.37	0.690
		Comfort	Male	2.07	0.639
			Female	2.47	0.650
		Enjoyment	Male	2.04	0.786
			Female	2.21	0.700
	Instrumental factors	Efficiency	Male	2.13	0.794
			Female	2.27	0.824
		Reliability	Male	2.04	0.706
			Female	2.27	0.780
		Size^a^	Male	2.21	0.870
			Female	2.32	0.828

a Size refers to the length, number and coverage of high occupancy vehicle lanes in a city.

**Table 4:** Survey factors and corresponding question numbers in part II of questionnaire.

Survey factors	Safety	Fairness	Comfort	Enjoyment	Efficiency	Reliability	Size
Corresponding question numbers	5	9	17	19	22	28	24, 25

## 2 Experimental design, materials and methods

High Occupancy Vehicle (HOV) lane is a special lane that permits only vehicles having at least two persons. An HOV lane is considered as a feasible method for improving transportation efficiency and it has attracted increasing research interest in developing countries such as China. Improved transportation efficiency gained from the HOV lane may also increase the Subjective Well-Being (SWB) of the drivers and passengers. To explore the public's SWB on HOV lanes, a questionnaire investigation from online survey was carried out in this study. Based on the study by Ettema et al. [1], a standard questionnaire was designed, shown in Appendix A. In the present study, we consider affective factors and instrumental factors as the components of SWB on HOV lanes; affective factors include safety, fairness, comfort and enjoyment, and instrumental factors include efficiency, reliability and size. An online survey was carried out on an online questionnaire website (www.sojump.com) between May and October, 2016. Totally 695 valid questionnaires were collected and processed. The demographics of these survey takers are shown in Table 1. The survey data are shown in Table 2, Table 3. The Factors in Table 2, Table 3 refer to Corresponding Question Number defined in Table 4. The values of Mean and Standard Deviation were computed from Part II of the questionnaire based on a scale of 5, with choice 1 = 5, choice 2 = 4, choice 3 = 3, choice 4 = 2, and choice 5 = 1.

## Appendix A Questionnaire

**Hello! This is a questionnaire about HOV lanes. All of information is anonymous and for academic research purposes only, so your personal privacy is promised. Please answer the followed questions truthfully and thank you for your cooperating.**

**Part I** The following questions are concerned with your Personal information.

**1. Gender.**

(1) Male (2) female.

**2. Age.**

(1) Below 18 (2) 18–30 (3) 30–45 (4) over 45.

**3. What kind of city scale are you from?**

(1) First-tier city (2) second-tier city (3) others.

**3. Main transportation methods.**

(1) On foot (2) Bus (3) Subway (4) Private car (5) others.

**4. What's your daily travel purpose?**

(1) Work (2) daily shopping (3) entertainment (4) picking up children (5) others.

**5. Have you heard or used HOV lane before?**

(1) Yes (2) No.

**Part II:** Have you saw the phenomenon that a private car with five seats carries one person only? Too many private cars make roads blocked while occupancy is low actually. High Occupancy Vehicle lane means a special lane which a vehicle with no fewer than two or more persons can use only. As always, HOV lane is clear while normal lanes beside it is blocked. In order to driving on HOV lane, sometimes there is a need to carpool with strangers and this behavior is encouraged by the government. Please answer followed questions.

**1. How is your mood in the past month?**

(1) Very good (2) Good (3) so-so (4) Bad (5) Very bad.

**2. Did you feel worried or anxious in the past month?**

(1) Never (2) Seldom (3) Sometimes (4) Often (5) Always.

**3. Do you satisfied with traffic conditions of your daily routines?**

(1) Strongly satisfied (2) Satisfied (3) Never mind (4) Not satisfied (5) Strongly not satisfied.

**4. What's your opinion on traffic jam?**

(1) Blame on overmuch private cars, while someone driving alone in peak time.

(2) It's a inevitably urban disease. (3) It's a hard problem to solve, but solve it as soon as possible.

(4) More lanes are required. (5) Other______________.

**5. Would you let strangers in your car for HOV lane?**

(1) Strongly agree (2) Agree (3) Never mind (4) Disagree (5) Strongly disagree.

**6. Would you accept an invitation from a stranger for HOV lane?**

**7. Do you think it is necessary that determine the routine before carpooling for HOV lane?**

**8. Do you agree that during carpooling, the driver changes the routine you determined before carpooling?**

**9. What's your opinion on deregulation as only one person driving a car on HOV lane?**

**10. Would you feel angry about a person driving alone in a traffic jam?**

**11. When you are driving a car alone in a traffic jam, while the HOV lane beside you is clear, should you allowed to use it?**

**12. Do you agree that ambulances or police cars drive on HOV lane?**

**13. Would you agree to pick up a stranger to use HOV lane without rewards?**

**14. Would you agree to charge the driver an appropriate fee for using HOV lane?**

**15. Would you try to avoid driving alone at peak time so you can use HOV lane?**

**16. Do you agree on the rule of passenger limit of HOV lane makes us more inconvenient?**

**17. Do you agree that HOV lane is a win-win situation while it conveniences yourself and help others?**

**18. Do you agree that HOV lane will improve traffic conditions and save social resources?**

**19. Would you feel happy when driving on HOV lane?**

**20. Do you think that HOV lane is a great idea?**

**21. Do you think that HOV lane do really help to reduce commute time?**

**22. Do you think that HOV lane will improve transportation efficiency of whole society?**

**23. Do you think changing normal lanes into HOV lanes will generate more blocked roads?**

**24. Do you agree that extended length of HOV lane is required in your city?**

**25. Do you agree we should increase the number of HOV lanes in your city?**

**26. Do you agree to that HOV lanes are widely adopted in your city?**

**27. Do you believe HOV lane can make you travel time saving?**

**28. Do you think that HOV lanes make you to go to destination reliably?**

**29. Do you think that HOV lane is a kind of effective and feasible solution to traffic jam?**

**30. What do you think that the promotion of HOV lanes will meet resistance?**

**31. What your overall view about HOV lane?**

(1) Strongly agree. It's an excellent idea. (2) Agree. It conveniences my transportation.

(3) Never mind. (4) Disagree. More lanes are required.

(5) Strongly disagree. It is a completely bad idea.

*****NOTE: Questions 6–30 have the same choices as:(1) Strongly agree (2) Agree (3) Never mind (4) Disagree (5) Strongly disagree. These choices are not shown above for the sake of brevity.

**Thank you for your participation.**
